# Learning and teaching of fluent musical note recognition: the visual perceptual perspective

**DOI:** 10.3389/fcogn.2025.1439439

**Published:** 2025-12-01

**Authors:** Yetta Kwailing Wong, Jiaqi Fion Fang

**Affiliations:** 1 School of Psychology, University of Surrey, Guildford, Surrey, United Kingdom; 2 Institute for Sustainability, University of Surrey, Surrey, United Kingdom; 3 Department of Educational Psychology, Faculty of Education, The Chinese University of Hong Kong, Shatin, Hong Kong SAR, China

**Keywords:** music education, expertise, music reading, perceptual expertise, perceptual learning, musical notation, music learning, visual recognition

## Abstract

Musical notation enables communications between composers, performers, music learners and music lovers. However, learning and teaching of fluent musical note recognition is often thought to be highly challenging. This paper aimed to summarize the current understanding of development of musical note recognition, explain its pedagogical bottleneck, and propose a pedagogical tool to address this problem. Review of the psychology and neuroscience literature identified eight psychological factors associated with fluent recognition of musical notes at both behavioral and neural levels. Many of the identified factors involve specialized visual perceptual mechanisms that are automatic, implicit and without conscious effort. Since classroom teaching heavily relies on verbal explanation, which cannot efficiently address these visual perceptual mechanisms, musical note recognition becomes difficult to teach and learn. We propose that visual perceptual training can serve as an innovative pedagogical tool to efficiently relax the visual bottleneck and enhance fluency in recognizing musical notes. We discuss why theoretically it works, the empirical basis for its effectiveness, its advantages, and potential concerns of adopting this tool by the music education community. In sum, visual perceptual training can directly facilitate development of fluency in recognizing musical notes in an efficient and personalized manner. This will encourage music exposure, learning and participation, and may therefore widely benefit the music learning community.

## 1 Introduction

Musical notation is the means of communication between composers, performers, music learners and music lovers. It allows composers to express what needs to be played and how, with which performers interpret composers' ideas and perform music. With the development of musical notational system, music has changed from a purely oral or play-by-ear tradition to incorporating a written culture (Kodály and Bónis, 1974). Nowadays, while music reading is not essential for music making (e.g., pop, jazz or folk music), music learners often spend a lot of time reading musical notes, especially for those trained in Western classical music.

The ability to recognize musical notes fluently is thought to be very helpful in music learning and development. For example, fluent readers are more likely to be immersed into a wide range of repertoire in different musical styles, and understand the intricate structure of complicated music pieces more easily (Stewart et al., 2003). Fluent reading also makes it easier to participate in diverse forms of collaborative musical activities since music scores help coordinate what each person should do in written, making it unnecessary to rely on one's memory for their parts. It is particularly helpful with large-scale ensemble, with complex pieces with which communication among musicians tends to become challenging, or when one is not provided with sufficient time for rehearsal. Consistently, fluent recognition of musical notes is important for many professional musicians to perform their job, and for amateur musicians to enjoy more rewarding and fulfilling musical life (Sloboda, 2004b).

In contrast, poor readers may struggle in music learning because figuring out each of the musical notes on the score could be difficult and slow (Stewart, 2005), and therefore their music exposure and development is likely to be more restricted. Through ear learning, poor readers can still enjoy music making and become professional and highly successful musicians, especially in genres that emphasize improvisation and performance. However, learning by ear is by nature more memory taxing and time consuming because one needs to learn by listening to each of the musical notes sequentially in accordance with their temporal order and memorizing them. Also, their learning is constrained by the setup of the recordings, e.g., whether the melodic and the accompaniment parts are separately recorded or whether one has the skill to identify their to-be-learned parts in the recordings with all parts combined. It therefore imposes a natural limitation on the learning of a large number of pieces and genres, especially those that are more lengthy, more complex, larger in scale, involve more parts, or with limited availability of recordings.

Unfortunately, learning musical note recognition is highly challenging (Lehmann and Kopiez, 2009). Anecdotally, it is common to hear that students find recognizing musical notes difficult, leading to some eventually giving up on music training (Mills and McPherson, 2006). Worst still, this skill is also difficult to teach. Teachers and expert musicians, as fluent readers themselves, admitted that they were not sure what to do to help students achieve fluent recognition because their teaching led to improvements in good-readers but not in poor-readers (Wolf, 1976)^1^. Indeed, some would explain away this pedagogical difficulty by describing musical note reading as an “inborn talent” that cannot be taught (Sloboda, 2004b; Wolf, 1976).

However, musical note recognition is a learned skill. One of the earliest forms of musical notation was found in 1400BC (Killin, 2018; Kilmer and Civil, 1986; Rankin, 2018; Wulstan, 1971), suggesting that musical notation was invented about 3500 years ago. Relative to the time scale of evolution, the invention of musical notation was too recent to be incorporated into our genetic codes, and therefore it is logically impossible to have any inborn neural mechanisms specifically dedicated to musical note recognition, similar to the case of letters and words (Dehaene, 2005). In other words, no musicians could be born with hard-coded neural substrates that prepare one for recognizing musical notes. Instead, music learners need to “teach the brain” to employ existing neural mechanisms to recognize musical notes through exposure and experience.

Given musical note recognition is a learned skill, it is important to understand what contributes to this pedagogical challenge. However, musical note recognition did not receive the attention that it deserves in the literature (Gudmundsdottir, 2010; Hodges and Nolker, 2015; Sloboda, 2004b). For example, major collections of research in music psychology discuss extensively on various topics related to music, including auditory perception, music cognition, performance, expertise and skill development, multisensory processing, emotion, the brain, language, evolution, special education, daily lives, etc. (Colwell and Webster, 2011; Deutsch, 1982, 1999, 2013; Gordon, 1971; Hodges, 2019; Patel, 2003, 2007; Peretz and Zatorre, 2003; Sloboda, 1985; Thaut and Hodges, 2019). In contrast, they do not have any chapter contributed to the discussion on musical note recognition (with the rare exception of Hodges and Nolker, 2015; Sloboda, 2004a). With scarce publications, not much is known about musical note recognition despite its importance (Gudmundsdottir, 2010; Sloboda, 2004b). As a result, we do not know much about what contritbues to the pedagogical bottleneck of learning and teaching of musical note recognition, and how to address it.

## 2 Purpose of this paper

This purpose of this paper is to take the first step to fill this research gap of addressing the pedagogical challenge of musical note recognition through three aims:

In Part 1 of the paper, we summarized the current understanding of development of musical note recognition, which is currently lacking in the literature, by reviewing the psychology and neuroscience literature. This led to identification of eight psychological factors associated with fluent recognition of musical notes.

In Part 2, we explained why musical note recognition is pedagogicallsy difficult based on the findings of the literature review in Part 1, highlighting how visual perceptual mechanisms complicate the development of this skill. In brief, we propose that fluent recognition of musical notes often involve specialized visual perceptual mechanisms that are automatic, implicit and without conscious effort. These make this skill impossible to explain through verbal communications in classrooms, directly causing the difficulty of learning and teaching of musical note recognition.

Finally, in Part 3, we propose that visual perceptual training can serve as an innovative pedagogical tool to efficiently relax the visual bottleneck and enhance fluency in recognizing musical notes. We discuss why theoretically it works, the empirical basis for its effectiveness, its advantages, potential concerns of adopting this methods by the music education community, and how adopting this method may widely benefit the music learning community.

## 3 Defining musical note recognition

In this paper, “musical note recognition” is defined by the *visual recognition* of musical notes in the Western musical notational system, i.e., the five-line staff. Visual recognition of notes is essentially a process of understanding the *visual shape* of the notes (e.g., the round dot, the stem, the position of the dot on the five-line staff, etc.), and the extent of understanding should be sufficient to enable one to differentiate the notes from other visually similar alternatives. For musicians, such visual understanding is often expressed in various forms of output, e.g., by motor execution of the musical notes on an instrument (i.e., music performance), imagining the sound of the musical notes (i.e., audiation, Gordon, 1999, 2007), verbal naming of the notes, or conceptual understanding of the music.

While these outputs are often the “end goals” of recognizing musical notes for musicians, it is important to note that visual recognition of the musical notes is a psychological process that is *separable* from these forms of output. For example, a visually impaired musician can be excellent in music performance, audiation, and conceptual understanding of music, etc., but they would find it highly difficult to understand the visual shape of the notes presented on a music score (e.g., Stevie Wonder and Andrea Bocelli who are blind and widely recognized musicians). Also, it is possible to train a person to be an expert in visually recognizing the shape of the notes without learning any of these forms of output (even though most people may question the purpose of such training) (Wong et al., 2019a). While visual recognition of musical notes is often strongly associated with all these forms of output in real-world music practice, it is important to acknowledge that they are separable psychological processes.

Even though defining “musical note recognition” as the visual recognition of musical notes may seem to be limited and narrow from the perspective of real-world and multimodal music practice, it is important to understand visual recognition of musical notes as a standalone skill and how it is associated with other musical skills. This can be illustrated by discussing the case of visual word recognition. Analogously, the “end goals” of visual word recognition are often to comprehend the text, read aloud the words, understanding the meaning, etc., and yet research work focusing on visual word recognition per se, as a standalone skill, is considered critical to understand word reading. In particular, many cognitive models are developed specifically to understand visual word recognition and how visual word recognition is related to other aspects of word reading (e.g., Davis, 2010; Grainger and Jacobs, 1996; Grainger et al., 2008; McClelland and Rumelhart, 1981; Norris, 2009; Norris and Kinoshita, 2012; Rumelhart and McClelland, 1982). Similarly, it is important to understand musical note recognition per se and how musical note recognition is related to other aspects of music learning and performance. Pedagogically, it is well possible that learning bottlenecks specific to the visual note recognition may occur in some of the music learners, leading to the frustration of learning and teaching (Mills and McPherson, 2006; Wolf, 1976). Hence, it is important to understand visual recognition of musical notes as a standalone skill, and how this skill can help explain and enhance music learning.

Musical note recognition is different from “sight-reading”. Sight-reading refers to the ability to perform music when one reads the music score for the first time and without practice (Fan et al., 2022). It is different from musical note recognition because musical note recognition does not involve any explicit motor execution of the music scores on any musical instruments, while “sight-reading” does.

In the literature, the term “music reading” has been used to refer to either “sight-reading” (Gudmundsdottir, 2010; Lehmann and Kopiez, 2009; Lehmann and McArthur, 2002; Mills and McPherson, 2006) or visual recognition of musical notes as defined in this paper (Burman and Booth, 2009; Drai-Zerbib and Baccino, 2014; Madell and Hébert, 2008; Puurtinen, 2018), and therefore we use the term “musical note recognition” and avoid using the term of “music reading” in the current discussion to minimize confusion.

The discussion below focuses on the *fluency* of recognizing musical notes visually rather than the basic identification of musical notes among beginners e.g., whether the pitch name of a note in the Western music scale is an “A” or “A#”. This is because most music learners can eventually learn to name and identify the notes with some effort or strategies, while many musicians find it difficult to achieve fluency in recognizing the notes (Green, 2017; Mills and McPherson, 2006; Wolf, 1976).

Lastly, each musical note carries both the pitch and rhythmic information. The focus of the current discussion is on the recognition of pitch of the notes. Visual recognition of rhythmic information is not at the center of discussion because they are often defined by salient visual features (e.g., stems, tails, black or white color of the dot, etc.). Empirical evidence has demonstrated that visual discrimination of notes based on rhythmic information can be achieved with similar accuracy and speed by musicians and non-musicians (Wong and Gauthier, 2010b; Wong et al., 2014), suggesting that rhythmic recognition of notes is not a major challenge in musical note recognition. Notably, visual recognition of notes based on rhythmic values should not be confused with rhythmic *execution* of notes. Rhythmic execution of notes requires conceptual understanding of the time value of the notes and motor execution of the notes on a musical instrument, and is a well-known challenge among music learners anecdotally. In contrast, visual discrimination of notes based on pitch information demonstrated a wide range of performance among musicians and non-musicians (Wong et al., 2021), demonstrating that this is likely be the key bottleneck of musical note recognition.

## 4 Defining music learners

In this paper, the term “music learners” refers to individuals who engage in learning music and who read Western five-line staff notation. This usage is intentionally broad, encompassing learners of different ages (children, adolescents, adults, and elderly), proficiency levels (from novice to advanced), and instrumental contexts (e.g., keyboard, string, wind). While most of the empirical evidence reviewed in relation to the psychological factors of musical note recognition (see below, Part 1) is based on studies with adults, many of these findings adopt a developmental perspective, demonstrating how the relevant effects gradually emerge from novice through intermediate to expert readers. This suggests that the underlying factors apply generally across proficiency levels. Furthermore, as discussed in Part 3, perceptual learning mechanisms have been shown to operate effectively in children, adults, and older adults, indicating that visual perceptual training is not only highly adaptable to individual needs but also developmentally general across different age groups.

## 5 Part 1: psychological factors for musical note recognition

Our review of the psychology and neuroscientific literature is performed by a literature search on Web of Science and Google Scholar using the terms “music reading”, “music note recognition”, “sight-reading”, “musical note”, and subsequent cited reference search on relevant papers. Relevant findings were selected and summarized based on the definition of musical note recognition (see above) and whether the findings are relevant to understanding of development of visual fluency in recognizing musical notes. For example, papers that focused on categorization of visual object perception or computational modeling of note recognition that did not speak directly to the development of visual recognition of musical notes were not included in the following discussion.

**Figure 1:**
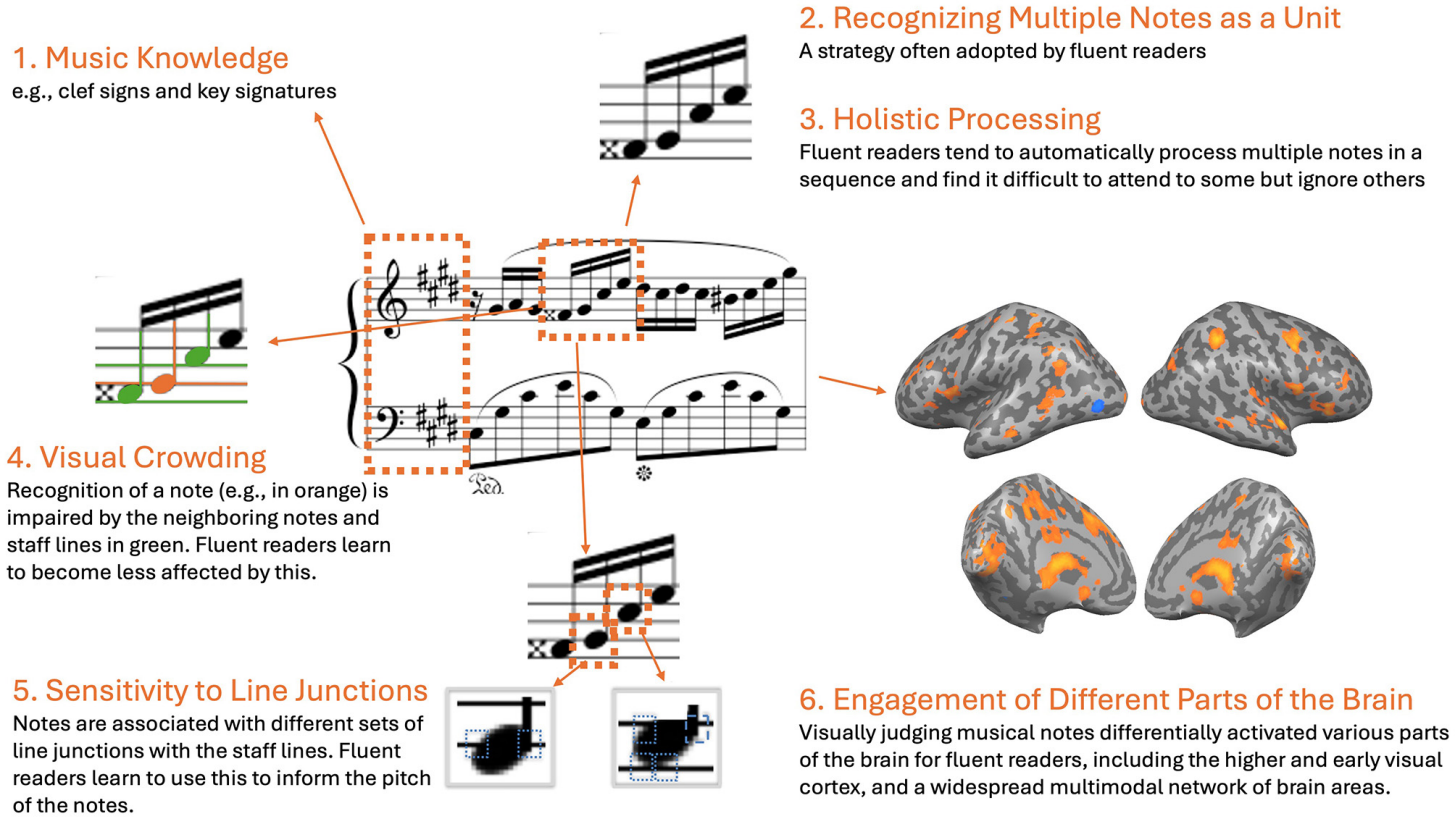
Summary of the psychological factors that can help differentiate good and poor readers of musical notes. The brain images illustrated the data from (Wong and Gauthier, 2010b).

The review suggests that eight psychological factors can help differentiate good and poor readers of musical notes (Figure 1). They include musical knowledge, reading multiple notes as a unit, holistic processing, alleviation of visual crowding, sensitivity to line junctions, engagement of higher visual cortex, engagement of a widespread multimodal neural network and of early visual cortex.

### 5.1 Musical knowledge

The influence of musical knowledge on musical note recognition is demonstrated by the phenomenon of proof-reading error and the research on eye-hand span. The proof-reading error was first reported by Boris Goldovsky, a distinguished musician and educator (Wolf, 1976). He described how his student, who was a poor reader, correctly performed a “G natural” as how it was written on the score. This surprised Goldovsky because the note must be a “G#” in the context of a “C#” major chord, and therefore he thought his student made a mistake at first. It turned out that the “G#” was misprinted as a “G” on the score, which has been correctly identified by the poor reader, but overlooked by Goldovsky and many other musicians in all the editions of the same piece available at that time (in Brahm's op76, no.2, measure 78; Wolf, 1976).

This observation was then confirmed by a subsequent experimental study (Sloboda, 1976). In this study, some visual notes in Baroque-Classical music scores were intentionally shifted by one step. Upon sight-reading, musicians made a significantly higher proportion of “errors” to these visually shifted notes than the unaltered notes, and the errors were nearly always what the notes should have been written originally. This finding confirmed that good music readers do not read the music score note-by-note as it is, but often infer the identity of the musical notes by musical knowledge.

Research on the eye-hand span also suggests that what musicians read from a music score depends on musical knowledge (Sloboda, 1974, 1977). Eye-hand span refers to the number of notes one can play on an instrument after the score disappears at an unexpected timing. Previous studies showed that the last note of the eye-hand span tended to coincide with the end of a musical phrase (Sloboda, 1977), and the size of the eye-hand span was affected by the complexity of the music (Lim et al., 2019; Rosemann et al., 2016). These suggest that musicians take into account the musical structure and content of a piece when they decide how much they read beyond the current time point. Consistently, adding white space between sub-phrases of the music scores led to fewer mistakes during sight-reading (Stenberg and Cross, 2019), suggesting that parsing music scores into sub-units according to musical knowledge facilitates the reading of music score.

The importance of music knowledge is also highlighted when good readers encounter difficulty in musical note recognition specifically with atonal and unfamiliar contemporary music (Wolf, 1976). One possible explanation is that this type of musical composition tend to have a relatively higher level of randomicity and the structure of the music tends to be less obvious. As a result, musical knowledge is not very helpful to predict the upcoming notes.

In sum, fluent music readers do not necessarily read what is exactly shown on the music score, and they do not always take in as much information as possible during reading. Instead, they apply their musical knowledge to predict and make educated guesses of the notes on the score, and flexibly determine how much information they preview on the score from the current time point based on music knowledge. This factor is particularly useful with music that follows a familiar structure.

### 5.2 Recognizing multiple notes as a unit

Intuitively, one can recognize musical notes more fluently by “recognizing multiple notes within a glance”, instead of recognizing each note one after another in isolation. This is consistent with the anecdotal report of expert readers, whose introspection suggested that reading performance can be enhanced by reading groups of notes simultaneously as a unit, a chunk, or a pattern (Wolf, 1976). Note that sometimes musical knowledge can help assign meanings to a “unit” such that the grouping of notes makes musical sense to readers, while at other times the grouping of the notes may appear relatively random, especially when the music piece is unfamiliar to the reader (e.g., during sight-reading) or composed in a less structured way (e.g., in contemporary styles). In this case, “a unit” or “a pattern” may simply refer to “several notes that are adjacent to each other”.

The idea of recognizing groups of notes simultaneously has a nice correspondence with the concept of eye-hand span and eye movement patterns of fluent readers. It has been well established that the size of the eye-hand span increases with sight-reading ability (Gilman and Underwood, 2003; Lim et al., 2019; Sloboda, 1974, 1977; Truitt et al., 1997). Also, better sight-readers tend to produce less and shorter fixations during reading, i.e., the period of time when the eyes stay on roughly the same position for acquiring new information (Goolsby, 1994; Penttinen et al., 2013; Truitt et al., 1997; Waters et al., 1997). These suggest that fluent readers can recognize a larger group of notes within shorter time during sight-reading.

### 5.3 Holistic processing

Experts often perceive visual objects in their expertise domain as wholes rather than as separable parts within the objects (Farah et al., 1998; Maurer et al., 2002; Young et al., 1987). This perceptual tendency is called “holistic processing” and is observed with many object categories including faces (Richler et al., 2012), dogs (Diamond and Carey, 1986), cars (Gauthier et al., 2003), words (Wong et al., 2019b), and Chinese characters (Wong et al., 2012). It is also observed with musical notes among musicians (Richler et al., 2011; Wong and Gauthier, 2010a).

In this study, participants were presented with two sequences of musical notes one after another, with each sequence containing four notes. All the notes were either connected with a straight line (i.e., 8^th^ notes) or disconnected (i.e., 4^th^ notes) within each pair of presented sequences. They were required to judge whether one of the notes (e.g., the 2^nd^ one from the left) were the same or different in the two sequences as accurately and as fast as possible. Compared with non-musicians, musicians found it more difficult to selectively attend to only one note in the sequence and ignore the other notes, even when they were explicitly told that the other notes were sometimes altered to interfere with their judgment. Results were similar regardless of whether the notes were connected or disconnected. These suggest that they learn to automatically and holistically attend to all of the notes within the sequences (Richler et al., 2011; Wong and Gauthier, 2010a). The more efficiently musicians can recognize musical notes, the more their judgment was influenced by the to-be-ignored notes, which was not observed among non-musicians (Richler et al., 2011; Wong and Gauthier, 2010a). These indicate that more fluent readers learn to develop a stronger tendency to holistically process musical note sequences.

Importantly, the note sequences used in this study were randomly generated with a computer algorithm, and therefore this perceptual tendency was unlikely driven by readers extracting musical meaning out of the sequences. The randomness of the presented notes makes this phenomenon different from simply grouping notes as a unit according to musical knowledge as discussed above. The automaticity of this effect also demonstrates that it does not simply stem from one's preference or selected strategy of “recognizing multiple notes as a unit” (Wolf, 1976). Instead, holistic processing of note sequences becomes more automatic and less suppressible when one becomes more fluent in recognizing musical notes.

### 5.4 Visual crowding

Visual crowding refers to the impairment of visual object recognition when the objects are surrounded by other visual elements (Levi, 2008; Pelli and Tillman, 2008; Whitney and Levi, 2011), such as letters, words and symbols surrounding each other during reading. It is regarded as a major limits of reading speed (Legge et al., 2007; Pelli and Tillman, 2008).

Visual crowding also affects musical note recognition (Wong and Gauthier, 2012). In this study, participants were required to judge whether a black dot was presented through a line or not (i.e., above or below the line). These stimuli resembled musical notes because the dot and the line were extracted from a real musical note. The crowded condition was created by adding extra dots adjacent to the to-be-judged dot or by adding extra lines on top of and below the to-be-judged dot.

In addition, participants were also required to judge whether a gap was added to the top or bottom of an open square, which were used as non-musical stimuli to control for general visual ability. The crowded condition was created by adding extra open squares adjacent to the to-be-judged square. It was expected that all participants would perform worse for the crowded condition than the uncrowded condition, and the question was the extent to which musicians and non-musicians were affected by crowding, and whether it differed with musical and non-musical stimuli.

Compared with non-musicians, expert musicians were less affected by visual crowding created by the extra visual elements surrounding the to-be-judged dot (Wong and Gauthier, 2012). This effect was only observed with musical stimuli but not with non-musical stimuli, suggesting that the observed effect could not be explained by musicians having better visual abilities in general. Also, more fluent music readers tended to experience less visual crowding (Wong and Gauthier, 2012). These suggest that musicians learn to better cope with the challenge of visual crowding specifically for musical notes with long-term experience in recognizing musical notes.

A subsequent training study further demonstrated that reduced visual crowding with musical stimuli is *caused* by visual experience in recognizing musical notes (Wong and Wong, 2016). After 8 h of visual perceptual training with musical note recognition, participants became more fluent in recognizing musical notes. Importantly, after training, participants also showed reduced visual crowding with musical stimuli but not with non-musical stimuli. These demonstrate that the reduced visual crowding with musical stimuli cannot be explained by any pre-selection differences among the musicians and non-musicians, e.g., individuals who perceive musical notes better tend to learn music because of their perceptual advantage and therefore become musicians. Instead, it is caused by one's perceptual experience and improved perceptual ability with musical notes.

### 5.5 Sensitivity to line junctions of notes

Line junctions are important cues of visual object recognition since removing them leads to impaired recognition of the objects, including common objects (Biederman, 1987), and letters and words (Lanthier et al., 2009; Szwed et al., 2009). In the Western musical notational system, the pitch of musical notes is defined by the position of the note on the five-line staff, which is in turn associated with different sets of line junctions (Wong and Wong, 2018). For example, when a musical note is on a staff line (e.g., a “E4” on the treble clef), there are two convex junctions, one on each side of the note. In contrast, when a musical note is between two staff lines (e.g., an “F4” on the treble clef), the line junctions become three concave junctions without any convex junctions. It is possible that picking up the information of line junctions of the notes is useful to inform the pitch of the notes, and therefore enhance fluency of note recognition.

By removing the small section of staff lines that were in touch with the dot of the notes, the junctions between the notes and the staff lines were also removed. Interestingly, this selectively impaired the note recognition performance of experts and intermediate music readers, while it did not affect performance of novice readers (Wong and Wong, 2018). The degree of performance impairment created by junction removal was predicted by one's fluency in recognizing musical notes, suggesting that more fluent music readers learn to develop higher sensitivity to the line junctions of musical notes.

### 5.6 Engagement of the higher visual cortex

To identify the neural regions that are engaged by recognition of musical notes, novice music readers and fluent readers were presented with single musical notes during functional magnetic resonance imaging (fMRI) (Mongelli et al., 2017; Sergent et al., 1992; Wong and Gauthier, 2010b). Visual stimuli with basic visual features matched with that of the notes were also used as a control condition so that the identified brain areas are specifically activated by musical notes instead of by visual stimulation in general, and to a larger degree among fluent readers than novice readers.

Results showed that multiple regions of the higher visual cortex are more activated by single musical notes among fluent readers (Mongelli et al., 2017; Sergent et al., 1992; Wong and Gauthier, 2010b). This is observed in the occipitotemporal cortex in both hemispheres, and corresponds well with the high functional specialization of the visual cortex, in which different areas in the visual cortex are preferentially activated by different object categories (e.g., faces, buildings, tools, letters and words, etc.,Grill-Spector and Malach, 2004). This preference in activation is further enhanced when participants develop perceptual expertise with the specific object domains (Gauthier et al., 1998; Moore et al., 2006; Op de Beeck et al., 2006; Wong et al., 2009; Yue et al., 2006).

### 5.7 Engagement of a widespread multimodal neural network

Fluent readers selectively recruit a widespread bilateral neural network when they recognize single musical notes (Mongelli et al., 2017; Sergent et al., 1992; Wong and Gauthier, 2010b). The network covers multimodal brain regions including early and late visual cortex, and auditory, audiovisual, somatosensory, motor, parietal and frontal areas. When more complex stimuli such as music sequences of several notes were used, a qualitatively similar but less robust neural network was engaged, again confirming that musical note recognition engages widespread multimodal network of brain regions (Nakada et al., 1998; Pantaleo et al., 2024; Proverbio et al., 2024, 2013; Wong and Gauthier, 2010b).

These results were observed when participants performed simple visual judgment with the stimuli without any explicit task demand on auditory, motor or semantic judgments (Mongelli et al., 2017; Sergent et al., 1992; Wong and Gauthier, 2010b). This demonstrates that the engagement of the non-visual areas of the neural network is relatively automatic. In a subset of brain regions in this neural network, the degree of specialized neural activity for musical notes was predicted by individual fluency in recognizing musical notes, suggesting that more fluent readers tend to automatically recruit more of this widespread multimodal neural network (Wong and Gauthier, 2010b).

### 5.8 Engagement of early visual cortex

The fMRI studies revealed that the early visual cortex was more activated by musical notes than visually matched stimuli among fluent readers (Wong and Gauthier, 2010b). This finding was further confirmed by electroencephalography (EEG) and event-related potential (ERP), which showed that fluent readers selectively engage the primary visual cortex bilaterally in the early ERP component called the “C1” as early as 40–60 ms after a single musical note is presented on the computer screen (Wong et al., 2014). Importantly, individuals who recognize musical notes more fluently also tend to engage the C1 more for musical notes, suggesting that the recruitment of the primary visual cortex is associated with fluent recognition of musical notes (Wong et al., 2014).

This finding of the early C1 is important and yet surprising because the primary visual cortex is known to be sensitive to basic visual features such as contrast, luminance and spatial frequency, but does not differentiate between meaningless visual noise and intact objects as long as they are matched with the basic visual features (Gilbert et al., 2001; Grill-Spector and Malach, 2004). Visual objects become differentiable only in later stages of visual processing, e.g., with the N170 (Luck, 2005). Consistently, other domains of visual perceptual expertise such as faces, cars, dogs, birds, and words, etc., engaged the N170 and not the C1 (Bentin et al., 1996; Gauthier et al., 2003; Maurer et al., 2008; Tanaka and Curran, 2001; Wong et al., 2005). The early timing of the C1 effect makes it impossible to be explained by feedback neural signals from higher visual cortex because it takes time to occur, and therefore it must be locally generated in the primary visual cortex (Luck, 2005). Why do fluent readers engage the primary visual cortex with musical notes then?

It is possible that the primary visual cortex helps with primitive analyses of the visual features of musical note stimuli, which in turn supports their fluent recognition among fluent readers. Interestingly, in a study in which non-musicians went through an intensive laboratory training that resulted in expert-like fluency in reading musical notes, only the amplitude of the N170 was increased for musical notes after training but not for that of the early C1 (Wong et al., 2019a). This suggests that the recruitment of the early C1 is not a result of visual perceptual expertise per se, but may involve additional non-visual factors such as long-term multimodal integration.

### 5.9 Summary of psychological factors for musical note recognition

In sum, fluent recognition of musical notes is associated with psychological factors. At the behavioral level, fluent readers use musical knowledge to guide their recognition of musical notes, including their prediction of the upcoming notes and how far they look ahead on the music score. They are aware of their tendency to read a larger group of notes as a unit. They tend to automatically process music sequences in a holistic manner, and learn to extract in detail the musical notes despite the visually crowded music scores. They also learn to develop higher sensitivity toward the line junctions between the note and the five-line staff. At the neural level, fluent readers engage a widespread multimodal network of brain areas when they simply see a single musical note, and they rapidly and selectively activate the primary visual cortex for processing musical notes as early as 40–60 ms when the neural signal first reaches the visual cortex. These suggest that fluent recognition of musical notes is associated with a range of knowledge, strategies and specific visual perceptual mechanisms.

## 6 Part 2: explaining the pedagogical difficulty with musical note recognition

When the above psychological factors associated with fluent recognition of musical notes are placed in the context of pedagogy, it becomes clearer why fluent recognition of musical notes is difficult to learn and teach. First, it is well-known that professional musicians with excellent musical knowledge can still struggle with recognizing musical notes fluently (Wolf, 1976). This indicates that musical knowledge, while helpful for recognizing musical notes, is not the key bottleneck. Also, while looking at a larger group of notes is commonly acknowledged to be helpful, it is not a strategy that poor readers can simply adopt because they find it impossible to look further ahead even if they want to (Wolf, 1976). In other words, simply sharing with struggling individuals musical knowledge and the strategy of looking at a larger group of notes is insufficient to help.

Interestingly, the rest of the identified psychological factors involve specialized visual perceptual mechanisms that are automatic, implicit and without conscious and deliberate effort, which is consistent with that engaged by expert behavior in general (Johansen and Palmeri, 2002). For example, most of us learn to recognize human faces well, but we are not aware of and therefore cannot verbally describe how we automatically integrate information across different parts of a face and process the face as a whole, rather than perceiving the eyes, nose and mouth separately (i.e., holistic processing of faces; Richler and Gauthier, 2014).

When fluent readers reflected on how they tended to read musical notes as larger units (Wolf, 1976), they did not discuss the automaticity of their tendency to read musical notes as larger units, as in holistic processing, and therefore they were unlikely aware of the fact that it would be difficult for them to try *not* to read musical notes as larger units. To our knowledge, these mechanisms, including the battling with visual crowding or attending to the line junctions of the notes, have not been discussed in the literature of music psychology. Instead, these are only recently revealed with careful measurements and controls in laboratory experiments.

The neural recruitment for fluent musical note recognition also supports the involvement of automatic and implicit perceptual mechanisms. First, a wide range of multimodal areas in the auditory, motor and somatosensory regions are recruited by a simple visual task of judging single musical notes, confirming the high automaticity in engaging the non-visual areas in the brain with musical notes even when it is not relevant to the task in hand (Mongelli et al., 2017; Sergent et al., 1992; Wong and Gauthier, 2010b). Also, the visual processes in the early visual cortex, including the primary visual cortex, are known to be implicit and non-verbalizable (Gilbert et al., 2001).

Importantly, the implicit and unconscious nature of these perceptual mechanisms makes it impossible for music educators to verbally explain to students how these mechanisms work, or what one can do to better engage with these mechanisms. In other words, the pedagogical difficulty of this skill is not the fault of music educators, e.g., with limited knowledge about music pedagogy, or insufficient motivation to help. Instead, most of the psychological factors involved in fluent recognition of musical notes, especially for the specialized visual perceptual mechanisms, are not verbally explainable. So what can be done to help students who struggle with developing these specialized visual perceptual mechanisms? Below, we discuss how visual perceptual mechanisms can be developed in response to task demands and environmental inputs through visual perceptual learning.

## 7 Part 3: visual perceptual training as an innovative pedagogical solution

### 7.1 Visual perceptual learning

Visual perceptual learning refers to the relatively long-term changes to the visual perceptual system that improves its ability to respond to the environment (Goldstone, 1998). It involves changes in how humans pick up, reshape, filter and process information from what we see in the physical world (Kellman and Garrigan, 2009; Sasaki et al., 2010). Visual perceptual learning supports a wide range of real-world tasks, including discriminating between similar words, recognizing the faces of our old and new friends, identifying a tumor from x-ray scans, detecting potentially dangerous items from air security checking, etc. (Bukach et al., 2006; Kellman and Garrigan, 2009; Sasaki et al., 2010; Watanabe and Sasaki, 2015).

Similarly, fluent musical note recognition involves visual perceptual learning, e.g., to identify and discriminate between similar musical notes or between similar note sequences. Empirically, fluent readers are about two times faster in visually discriminating between highly similar note sequences than non-experienced readers (Wong and Gauthier, 2010a; Wong et al., 2021).

Notably, visual perceptual learning happens even when the visual signal is too weak to enter one's awareness and is presented in a task-irrelevant manner (Watanabe et al., 2001). In other words, the visual perceptual system is capable of sharpening its information processing and representation in response to the visual environment in an unintentional and unconscious manner. Hence, providing the appropriate visual stimulation and experience is the key to induce changes in the visual perceptual system.

### 7.2 What is visual perceptual training?

Here, we propose that visual perceptual training—inducing perceptual learning using explicit and specifically designed training protocols—can be used as an innovative pedagogical tool for improving the learning and teaching of fluent musical note recognition. Visual perceptual training is a well-established way to efficiently improve visual object recognition (Gauthier et al., 1998; Jiang et al., 2007; Moore et al., 2006; Op de Beeck et al., 2006; Weisberg et al., 2007; Wong et al., 2009, 2011; Wong and Wong, 2016; Yue et al., 2006). It typically adopts computer programs to present stimuli (e.g., images and/or sounds) many times to the participants, and require participants to judge and respond to the stimuli according to the task designs. By a careful selection of the stimulus sets, tasks, and course of training progression, the required judgment becomes more and more challenging, which pushes the visual system to alter its information processing and representation and therefore leads to desired improvements in the targeted domains of perceptual skill.

### 7.3 Effectiveness of visual perceptual training in general

Visual perceptual training works well in different populations including normal adults (e.g., Gauthier et al., 1998), children (e.g., Frank et al., 2021), individuals with visual impairments (e.g., Hussain et al., 2012), and the elderly (e.g., Polat et al., 2012). It works well with different objects such as faces (McGugin et al., 2011), words (Xue et al., 2006), and novel artificial objects created with computer programs (Gauthier et al., 1998; Moore et al., 2006; Op de Beeck et al., 2006; Wong et al., 2009; Yue et al., 2006). It is effective even when the to-be-learned visual signal is task-irrelevant and/or presented in an unconscious manner (Tsushima et al., 2006; Watanabe et al., 2001; Wong et al., 2011). It can bring about large-scale changes in the brain within 8–10 h of training (Gauthier et al., 1999; Wong et al., 2009). These demonstrate that the effectiveness of perceptual training is highly generalizable to different populations, age groups, stimuli and contexts.

### 7.4 Effectiveness of visual perceptual training with musical note recognition

It has been demonstrated that visual perceptual training can efficiently enhance fluency in recognizing musical notes. In a laboratory training study, participants briefly saw a four-to-five note sequence, and were subsequently required to select this sequence among a highly similar distractor sequence (Wong and Wong, 2016). The training progressed by gradually reducing the presentation time of the first sequence based on the performance of the participants. After 8 h of training, intermediate-level music readers recognized the four-to-five note sequences faster by 44.1% (Wong and Wong, 2016). With 10–26 h of a similar perceptual training, music novices attained a high level of fluency with recognizing four-to-five note sequences, which was comparable to that measured with real-world experts who have typically spent more than 10 years in formal musical training (Wong et al., 2019a). The demonstrated effectiveness of perceptual training echoes well with the reported success in the use of tachitoscope and computer-aided instruction in research in music psychology, which also used briefly presented musical notes during learning (Hodges and Nolker, 2015).

### 7.5 Comparing visual perceptual training with traditional pedagogical methods

In music education, traditional pedagogical approaches often emphasize learning other skills before learning to read musical notes, and embed the development of musical note recognition in broader multimodal learning activites. For example, Dalcroze engages students through eurhythmics, integrating rhythmic movement and kinesthetic awareness in teaching musical expression before introducing note recognition (Jaques-Dalcroze, 1921). For Dalcroze and Kodály, musical note recognition can be introduced in solfège course through ear training, sight-singing, and learning musical knowledge (Ittzés, 2004; Jaques-Dalcroze, 1921; Kodály and Bónis, 1974). Orff promotes improvisation and self-created graphical notation, enabling learners to internalize musical structure prior to engaging with conventional notation (Orff, 1977, 1982); Suzuki emphasizes listening and memorization through imitation, introducing note recognition only when pieces exceed auditory or memory constraints (Suzuki, 1992, 1998). Gordon's model centers on audiation, i.e., the internal hearing of music, which suggests students to understand music patterns meaningfully and develop instrumental readiness before starting to read musical notes (Gordon, 1999, 2007). When one is ready, meaning of musical notes is acquired through a learning sequence of music vocabularies, symbols, writing, improvisation, and their integration (Gordon and Woods, 1990; Walters and Taggart, 1989; Zheng, 2012).

While these methods are well-established and widely embraced pedagogically to provide valuable and holistic experience to learners, they do not discuss the development of fluent visual recognition of musical notes as a standalone skill separable from performance, audiation, or sight-reading—the definition of musical note recognition adopted in the current discussion. Blending multiple skills during learning, especially before mastery of each of the foundational components, may hinder effective learning (Bloom, 1968). For example, a sight-reading exercise requires students to read musical notes, listen and perform well simultaneously during the first time of reading (Sloboda, 1974; Waters et al., 1998). Students may struggle to integrate these skills during sight-reading when each of these skills is somewhat a challenge (Lehmann and Kopiez, 2009). Also, when a student does not perform well on this learning activity with multiple task demands, it may be difficult for teachers to isolate which skill(s) needs help because the demands share the same assessment outcome (Wong et al., 2021). These demonstrate the need to consider pedagogical tools other than the traditional approaches.

For learners who are working to develop fluency with recognizing musical notes, their learning may benefit from learning tools that focus specifically on development this visual skill first, before challenging them by combining multiple tasks together. Visual perceptual training can serve this purpose by strengthening this fundamental skill. In essence, visual perceptual training is not intended to replace traditional methods but to augment their efficacy by building a solid perceptual foundation that facilitates subsequent multimodal integration. Building on this groundwork of fluent note recognition, learners will then be enabled to better integrate audiation, sight-reading and performance.

### 7.6 Advantages of visual perceptual training

There are several advantages of using perceptual training as a pedagogical tool for musical note reading. First, it is readily adaptable to cater for the learning needs of different readers. For example, for those who read music scores composed of one-staff systems (e.g., flute and violin players), the training can focus on increasing the number of notes presented horizontally on each stimulus, i.e., expanding the horizontal visual span. For those who read two-staff systems (e.g., pianists), the training can aim at expanding both the vertical and horizontal visual span gradually such that learners can learn to capture notes in both dimensions without moving their eyes. Other parameters, including the number of notes on each stimulus, the presentation speed, the overall size of the stimulus, whether ledger lines are included, etc. can easily be adjusted to calibrate the difficulty level of the training to cater for the diversified needs of the learners.

Second, the progression of training can be individualized such that the learners can proceed faster or more slowly according to their ability and degrees of improvement. With a computer program, this can be automatized such that faster learners can move to more challenging levels without wasting their time on beginner's materials. Also, the time taken and accuracy for each participant's response can be precisely recorded and analyzed. These help titrate the difficulty of the training trials to appropriate levels and understand the bottleneck of further improvement.

Third, visual perceptual training is often gamified, making it a fun and motivating learning experience. A common way to gamify perceptual training is to introduce a number of levels that learners need to pass, during which they collect various tokens as reinforcement (Wong et al., 2020; Wong and Wong, 2016).

Lastly, visual perceptual training can be performed without extra manpower, supervision or teachers' guidance, making it a cheap and accessible learning tool to complement in-class learning. These characteristics can directly lower the cost and reduce the class time required to improve this skill, which in turn improve the equality and inclusiveness of music learning for the disadvantaged and those with geographical constraints.

### 7.7 Potential concerns of visual perceptual training

While visual perceptual training is an effective way to enhance fluency in recognizing musical notes, music educators may be concerned about its appropriateness for music students because of several reasons.

#### 7.7.1 Improving visual skills in isolation and out of context

First, one may question the meaningfulness of “recognizing” musical notes in an abstract manner without linking them to appropriate forms of output such as performance, audiation and verbal naming of the notes—the typical “end goals” of reading musical notes for musicians. Also, training visual perceptual skills in isolation of other musical abilities seems to be at odd with the holistic approach commonly endorsed in music education that emphasizes the integration of multiple modalities during music learning (McPherson et al., 2016).

It might be helpful to think about visual perceptual training as additional exercises targeting on fundamental musical skills that complement music training. Similar to technical exercises devoted to finger dexterity, and listening exercises to understanding of rhythmic patterns or chord progressions, visual perceptual training helps develop specialized skills that can be improved more efficiently by specific and separate practices.

Importantly, during normal music training activities, students are still required to translate musical notes to musically meaningful outputs and integrate information processing in multimodal modalities. These on-going training will help students place what they learn during visual perceptual training into the appropriate musical context and use their knowledge holistically.

#### 7.7.2 Learning note recognition inappropriately may hurt music development

Second, music educators are often concerned that learning to read musical notes inappropriately would hinder students' development. They propose that musical note recognition should be learned *after* one has developed other basic skills, such as eurhythmy (Jaques-Dalcroze, 1921), considerable improvisational skills (Orff, 1977, 1982), basic performing skills through ear learning and memorization (Suzuki, 1992, 1998), or instrumental readiness with considerable understanding of tonal and rhythmic patterns (Gordon, 2003, 2007). According to the “sound before sign” approach, children should develop sufficient musical knowledge, sensory experience and/or motor experience before learning to read musical notation (McPherson and Gabrielsson, 2002).

A common concern underlying these suggestions is that focusing too much on visual recognition of musical notes may lead to drawback in musical development (McPherson and Gabrielsson, 2002). For example, it may compromise the development of the intimate feel of musical meaning of the sound or lead to some students executing the notes on the scores mechanically like a robot. Also, since recognizing musical notes can be highly frustrating for some, students may easily get demotivated, which affects their learning progress.

The above concern may be explained by the challenge of developing fluency in recognizing musical notes using traditional methods. When students are consumed with figuring out what note it is on the score, they are left with little cognitive resources for other aspects of information processing, such as audiation, motor planning, and emotion expression of the music. In other words, the potential undesirable drawbacks mentioned above are likely a consequence of experiencing *difficulty* in musical note recognition, rather than musical note recognition per se. In this case, visual perceptual training may actually help students avoid these undesirable drawback by directly enhances fluency in recognizing musical notes. With a relaxed bottleneck in visual recognition, it may be worth revisiting whether the above concerns would still exist.

#### 7.7.3 Suitability of visual perceptual training to different music learners

Third, the learning needs of music learners can be highly different. For example, children, adolescents and adult learners tend to have different developmental needs in visual perception, cognition and other educational needs (Best and Miller, 2010; Frank et al., 2021). Also, learners of different music instruments may also have different needs, such as keyboard players reading multiple staves of musical notes, and string players integrating symbols of articulation and fingering with musical notes. These pose a question on the suitability and effectiveness of visual perceptual training as a universal pedagogical tool.

Despite these differences, we argue that visual perceptual training remains a broadly applicable pedagogical tool. First, research has shown that visual perceptual learning is effective across a wide range of ages and backgrounds, from children and adolescents to adults and the elderly (Frank et al., 2021; Gauthier et al., 1998; Polat et al., 2012), and from typically developing individuals to those with special needs such as visual impairments (Hussain et al., 2012). Prior effective perceptual training with musical note recognition also covers both musical notes spanning one staff and two staves (Wong et al., 2019a; Wong and Wong, 2016). These suggests that, regardless of the developmental or instrumental differences among music learners, the fundamental principle of enhancing visual perceptual fluency through targeted training should still be effective. As discussed above, visual perceptual training is highly adaptable and can be readily tailored to the specific needs of learners, including different number of staves, stimulus size, presentation speed, or inclusion of ledger lines. Integration of articulation and fingering symbols in string and wind instruments can also be included flexibly. Together, these characteristics support the notion that visual perceptual training is not a one-size-fits-all approach, but a flexible framework that can accommodate the diverse needs of music learners across instruments, ages, and abilities.

## 8 Conclusion and future research

Apart from musical knowledge and preferred strategies, fluent recognition of musical notes involves specialized visual perceptual mechanisms. These mechanisms are automatic, implicit and do not require conscious effort, making it impossible for music teachers to verbally explain how they attain fluent recognition or to point out what students can do to enhance their fluency in recognition. These directly cause the difficulty of learning and teaching of fluent musical note recognition.

Empirical evidence has demonstrated how a few hours of visual perceptual training can efficiently relax this visual bottleneck for recognizing musical notes. With this innovative and powerful pedagogical tool, one may speed up learning progress, increase interest and motivation in learning, enhance the breadth and depth of music exposure, and encourage participations in different types of musical activities. The training protocol can also be personalized to cater for individual learning needs. As a result, music learning may widely benefit from this method. The music education community should consider incorporating visual perceptual training as a standard component of music learning.

Importantly, the present discussion is intended as a *proof of concept* rather than a prescriptive curriculum: the exact form of implementation will depend on how software and training platforms are designed for specific age groups, instruments, and classroom contexts. What we emphasize here is the potential of this approach to complement established pedagogical traditions by strengthening the visual foundation of note reading, thereby enabling multimodal integration in subsequent learning.

Given this innovative pedagogical method to relax the visual bottleneck of learning music, future research should empirically investigate the extent to which adopting this method may bring advantages to music learners, e.g., to improve efficiency of learning, reduce frustration, and to enhance the long-term learning outcomes of musical training. Future work should also empirically examine to what extent the “sound before sign” approach is a more beneficial way of learning music, and to what extent learning to read musical notes early in musical training may benefit or hinder music development. In addition, applied studies in real educational contexts will be essential for determining the most effective ways to integrate visual perceptual training alongside existing teaching practices.

## Footnotes

1 This paper discussed “music sight-reading”, which is conceptually differentiable from musical note recognition because music sight-reading requires one to perform the musical notes with an instrument while musical note recognition does not (see discussion on “defining musical note recognition”). However, this specific paper focused on the experience of professional musicians with comparable musical knowledge and abilities in learning, remembering and performing music, and therefore the difference between good and poor sight-readers was focused on their differences in visual recognition of the notes (see discussion on p164-168, Wolf, 1976).
